# UET175: EEG dataset of motor imagery tasks in Vietnamese stroke patients

**DOI:** 10.3389/fnins.2025.1580931

**Published:** 2025-06-17

**Authors:** Chau Ma Thi, Hoang-Anh Nguyen The, Kien Nguyen Minh, Long Vu Thanh, Hieu Nguyen Dinh, Nhu-Y Huynh Thi, Thanh-Huong Ha Thi, Trong-Nghia Hoang Tien, Doan-Truc Au Dao, Kim-Long Nguyen Hoang, Vy Huynh Kha, Tuyet-Linh Le Hoang

**Affiliations:** ^1^Human Machine Interaction Laboratory, University of Engineering and Technology, Vietnam National University Hanoi, Hanoi, Vietnam; ^2^Vietnam - Korea Institute of Science and Technology, Ministry of Science and Technology, Hanoi, Vietnam; ^3^Department of Neurology, Military Hospital 175, Ho Chi Minh City, Vietnam; ^4^School of Biomedical Engineering, International University, Vietnam National University Ho Chi Minh City, Ho Chi Minh City, Vietnam

**Keywords:** EEG signal, motor imagery, stroke patients, EEG dataset, brain-computer interface

## Introduction

Brain-computer interface (BCI) systems have garnered significant attention in clinical and research settings since the late 20th century. Using brain signals, BCI systems allow users to interact with machines without the need to move their limbs, making them particularly suitable for rehabilitating patients with severe neuromuscular impairments (Daly and Wolpaw, 2008; Triponyuwasin and Wongsawat, 2014; Muñoz et al., 2014). In particular, BCI systems based on electroencephalogram (EEG) signals are the most widely used, due to their low cost, noninvasive nature, and readily available hardware for both commercial and medical applications (Han-Jeong Hwang and Im, 2013). In the realm of post-stroke rehabilitation, Motor Imagery (MI)-based BCI systems have been a primary focus for over a decade (Cervera et al., 2017). These systems decode and classify neuronal signals associated with the process of imagining a movement, without any actual physical execution, to operate output devices or generate feedback in various forms, such as visual and auditory cues. Through training and neural plasticity, patients can link feedback to specific movements, thereby reinforcing their neuromuscular connections and gradually regaining control over their partially paralyzed limbs.

Rapid advances in neuroscience and computer science have significantly improved methodologies and environments for MI-BCI experimentation and clinical trials. Feedback mechanisms have progressed from merely displaying visual prompts (Triponyuwasin and Wongsawat, 2014) on computer screens to creating immersive virtual environments that can be experienced through virtual reality (VR) headsets (Vourvopoulos et al., 2016; Lupu et al., 2018). Additionally, other forms of feedback, such as electrical stimulation (Lupu et al., 2018) and vibrotactile feedback (Vourvopoulos et al., 2016), have been explored, producing meaningful results. In terms of MI task classification, the primary approach has involved using spatial filters such as Common Spatial Pattern (CSP) for feature extraction, combined with effective machine learning algorithms such as Linear Discriminant Analysis (LDA) or Support Vector Classifier (SVC) to identify the correct MI class for the current session (Ortner et al., 2012; Muñoz et al., 2014). Recently, researchers have turned to deep learning to address classification challenges in EEG data (Hossain et al., 2023), with notable state-of-the-art models including EEGNet (Lawhern et al., 2018), EEGTCNet (Ingolfsson et al., 2020), ATCNet (Li et al., 2024), and EEG-DBNet (Lou et al., 2024). Additionally, transformer-based architectures, such as SMT (Luo et al., 2023) and ITNet (Niu et al., 2024), have demonstrated promising capabilities in EEG analysis.

The models mentioned above have achieved commendable offline testing results on publicly available standardized datasets used for BCI competitions. The data sets used most extensively in this category include the BCI Competition IV-2a dataset (Brunner et al., 2024a), the BCI Competition IV-2b dataset (Brunner et al., 2024b), and BCI Competition III (Blankertz et al., 2006). However, these datasets, along with most other published ones, such as Kaya et al. (2018), the PhysioNet dataset (Schalk et al., 2004) and the High-Gamma Dataset (Schirrmeister and Ball, 2017) focused on MI-EEG, consist solely of recordings from healthy volunteer subjects, rather than actual patients suffering from post-stroke motor complications. Furthermore, these existing databases have limitations, such as short recording durations and low sample sizes. Recently, in 2024, a new dataset featuring 50 stroke patients was released by Liu and Han (2024). However, all of these examples were recorded using generally accepted medical-grade equipment, characterized by high sampling frequency, improved resolution, and signal stability. To create more accessible systems on a commercial scale, it is essential to use data recorded with commercially available devices to train machine learning models, thereby improving performance in clinical trials.

In this study, we introduce the UET175 database, which features a comprehensive collection of electroencephalogram (EEG) signals collected from 30 post-stroke patients at Hospital 175 in Ho Chi Minh City, Vietnam (Hospital 175), during the time-frame from October 2024 to January 2025. Our primary goal is to develop a standardized protocol for EEG data acquisition that guarantees both consistency and reliability in various research applications.

This database specifically emphasizes brain signal data related to motor imagery tasks, which are essential for advancing the development of a VR-BCI system designed to improve stroke rehabilitation outcomes. Furthermore, the data will contribute to the progress of other BCI systems based on motor imagery.

By making this database publicly accessible, we aim to foster research collaboration and provide invaluable resources for the scientific community interested in investigating brain signal data for both clinical and technological applications. The database can be accessed via the following *https://github.com/nmk-k66-uet/UET175.git*.

## Method

The data acquisition experiment described in this study was approved by the Institutional Review Board for Human Research at the Dinh Tien Hoang Institute of Medicine, operating under the codes IRB-VN02010 from the Vietnam Ministry of Health, and IRB00010830 and IORG0009080 from the U.S. Department of Health and Human Services. This study received the operating code IRB-2206 and was conducted according to the guidelines established by the Dinh Tien Hoang Institute of Medicine. Written informed consent was obtained from all participants, including stroke patients or their legal representatives, to allow experiments and the publication of their data. The methods outlined here complement a detailed description of the results derived from this dataset.

To recruit participants for the study, a notice inviting voluntary participation was widely disseminated. After clearly explaining the purpose of data acquisition and obtaining informed consent, the research participants (referred to as subjects) signed a commitment to participate with the principal investigator. For post-stroke patients who volunteered, neurologists from the Clinical Neurophysiology Unit of Hospital 175 conducted assessments to select individuals for data acquisition using NIHSS scale. Participants were then chosen based on specific inclusion and exclusion criteria. The inclusion criteria required patients with mild to moderate stroke (either ischemic or hemorrhagic) and an NIHSS score of 15 or less, who could participate after the acute phase. To ensure a smooth recording session, patients needed to retain the ability to comprehend spoken, written, or visual instructions and have a stable neurological condition. Conversely, the exclusion criteria eliminated individuals with motor function impairments not related to stroke, as well as those with severe visual or hearing deficits. Patients with hemispatial neglect, a history of epilepsy or uncontrolled seizures, severe conditions affecting the lungs, kidneys, liver, or heart, significant circulatory disorders in the limbs, an inability to sit independently for 60 min, or cognitive impairments that limited their ability to understand and follow medical instructions were also excluded. To further assist in analyzing the patients' conditions, prior to starting the trial, the patients will be additionally evaluated for motor function and their ability to perform daily activities based on the Oxford (in this case, the doctors performed an evaluation of muscle strength in the hand) and Modified Rankin (mRS) Scale.

In this study, the data recording process involved 30 volunteered post-stroke patients. Participants were identified solely by their aliases, starting from “ID01” to “ID30.” The data was recorded in a specialized medical environment designed for EEG signal recording at Hospital 175. Technicians met with each patient every 3–7 days to conduct recordings based on their health status and convenience, with each session lasting ~14–18 min, excluding the setup time for the EEG recording device.

**Figure 1:**
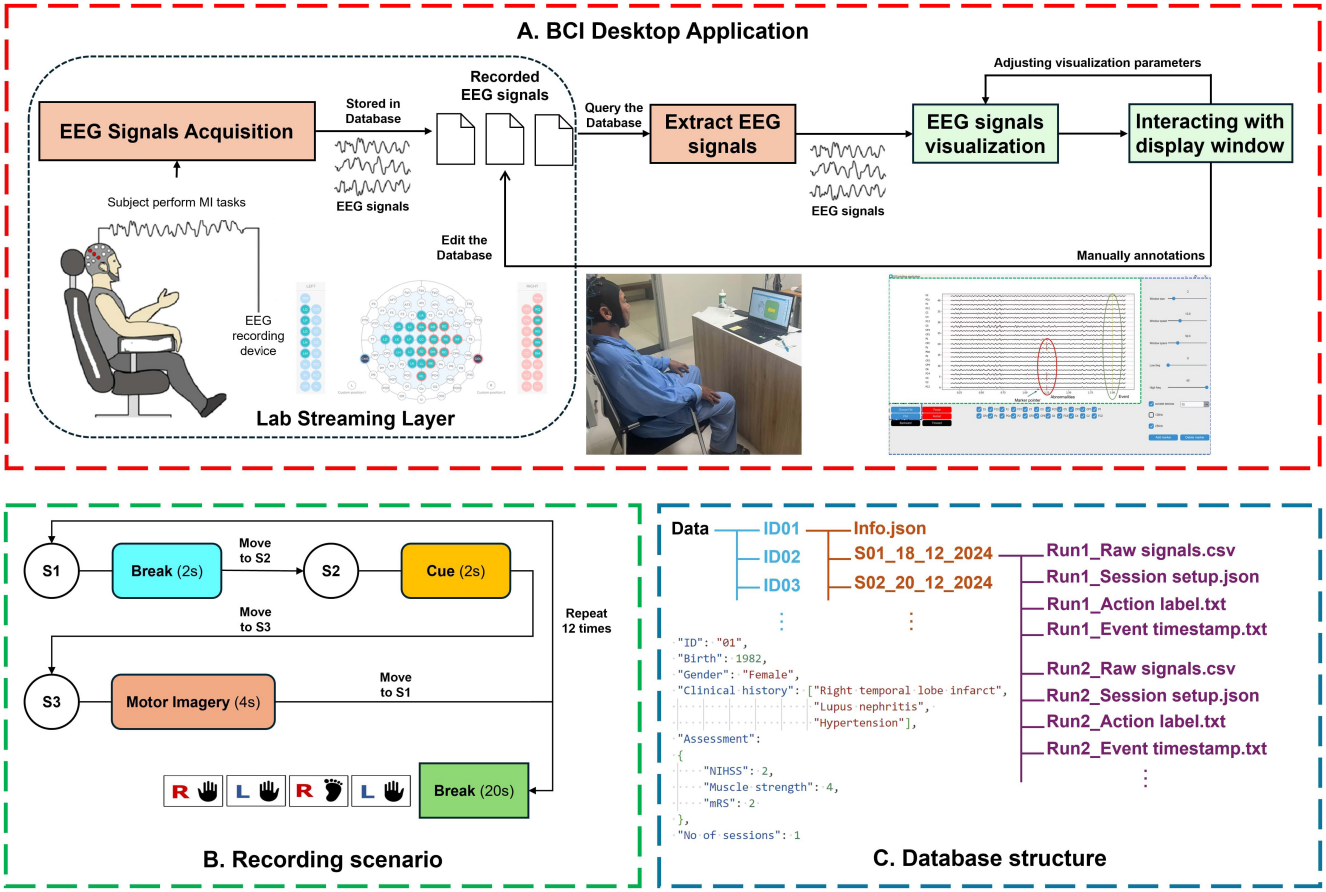
Overview of data acquiring process. **(A)** The process of recording EEG signals using the desktop application; **(B)** the recording scenario; **(C)** the dataset structure, data is organized by patient (blue) and then by session (orange), each session contains multiple runs (purple).

We recorded EEG data using an Emotiv EPOC Flex device, which utilizes EPOC+ technology. This system features a controller with a 128 Hz sampling rate and connects to a computer via Bluetooth or wireless USB. To enhance participant comfort, we employed a saline sensor version with 34 wired sensors, adhering to the international 10–20 standard for flexible sensor placement. Certain electrode positions, particularly those near the motor cortex, demonstrated clearer MI features. A research by Altaheri et al. (2023) indicates that reducing the number of EEG channels can maintain prediction accuracy while simplifying setup, expediting data processing, and conserving memory. We selected 22 channels near the motor cortex from the available 32, along with two reference electrodes. This configuration resembles the electrode montage used in the BCI Competition IV-2a dataset (Brunner et al., 2024a) (Figure 1A). The EEG data is transmitted using the Lab Streaming Layer (LSL) protocol, allowing for synchronized data acquisition. Key configuration settings include: Data stream—EEG, Sampling frequency—128 Hz, Data format—Double, and Transmit type—Sample. Before data transmission, the Emotiv device incorporates a 5th-order Sinc filter along with a bandpass filter ranging from 0.2 to 45Hz to effectively minimize noise and artifacts while preserving the integrity of brainwave signals.

### Recording application

EEG data were acquired using a BCI desktop application (Figure 1A) running on an Acer Aspire A315-57G laptop. Developed by our research group, this software enables automatic recording and synchronization between the recording scenarios and EEG signals. Users can connect to the EEG device and input information about the volunteer subject and session, which is then stored in our database. The recording function allows users to set up scenarios by selecting predefined options or creating custom actions with specified durations. Additionally, the module can automatically generate a video with visual and auditory cues for the subject, which can be played independently as a preview or simultaneously with the EEG recording.

Moreover, the application includes a visualization tool designed for analyzing EEG signals recorded during sessions, particularly in the time domain. Developed in collaboration with the aforementioned neurologists, it offers features such as adding time markers, adjusting signal speed, and selecting frequency bands for detailed analysis. Users can access data files from previous sessions and display EEG data within the app, allowing for real-time adjustments of display parameters, including window size, signal spacing, frequency bands, channels, and filtering options (Wavelet denoising or Bandpass filter). Users can also manually label channels based on timestamps of interest and save these annotations to the database. Considering that response times vary from one subject to another and from one instance to another, we assess whether a trial qualifies as a MI task based on the Event-Related Desynchronization percentage (ERD%—The proportion of power reduction in the EEG signal when compared to a baseline period) (Pfurtscheller and Lopes da Silva, 1999). For each trials, we identify a 1-second signal segment where the ERD% reaches at least a threshold of **T%** compared to the resting phase and store this information in a timestamp file. This segment is extracted from the final 0.5 s of each cue until the end of the MI task. Trials without at least one suitable signal segment are considered too noisy and will be rejected. From our experiment on data gathered at Hospital 175 and in our laboratory, setting the threshold **T** to **30%** filters out most of the data collected outside the hospital environment, which is considered significantly noisier, while allowing the majority of the remaining dataset to be retained.

After each recording session, four files are generated: *Raw Signals File, Session Setup File, Action Label File*, and *Event Timestamp File*.

### Recording scenario

Figure 1B illustrates the scenario used for data acquisition. In step 1, at the start of an action, the recording app plays a notification sound, prompting the patient to relax and loosen their body for 2 s. In step 2, the system displays a prompt image along with a notification sound corresponding to one of four MI tasks: lifting the right arm, lifting the left arm, lifting the right leg, or lifting the left leg, which lasts for 2 s. In step 3, the patient imagines the corresponding movement suggested by the prompt for 4 s. This process repeats 12 times (three times for each task mentioned in step 2) in each run, followed by a 20-s resting period between runs.

### Data directory structure

The dataset was organized using a hierarchical Unix-style filetree structure (Figure 1C). The main directory, labeled “**Data**”, contains 30 subdirectories, each of which named with a patient's ID. Within each patient directory, there is an Patient's info (**.json**) file as well as one or more subdirectories named based on the session number and date of the individual recording sessions. The patient's info file contains some detailed information about an individual patient, including a unique ID assigned to the patient; the patient's year of birth and gender; a list of medical conditions, diagnoses from the Hospital 175 database; various medical assessments, such as NIHSS, Muscle Strength measured in hand, mRS score; and the number of EEG recording sessions. Finally, each session directory includes one or more runs, each of which consists of a Raw signal (**.csv**) file, a Session setup (**.json**) file, an Action label (**.txt**) file, and an Event timestamp (**.txt**) file. All four files are named based on the run number to identify each run.

#### Raw signal file

- Format: CSV
- File name: run number_Raw Signals
- Content: contains signals from electrodes, with each column representing one electrode's data throughout the recording. Electrode order matches the EmotivPro app settings.

#### Session setup file

- Format: JSON
- File name: run number_Session setup
- Content: duration of each neurological task, the session includes a calibration block and repetition count of the scenario.

#### Action label file

- Format: TXT
- File name: run number_Action label
- Content: sequence of numbers representing actions in the scenario, ordered based on the JSON setup file.

#### Event timestamp file

- Format: TXT
- File name: run number_Event timestamp
- Content: list of MI task timestamps, adjusted for patient response time based on ERD and excluding any noisy trials that were rejected.

## Result

**Figure 2:**
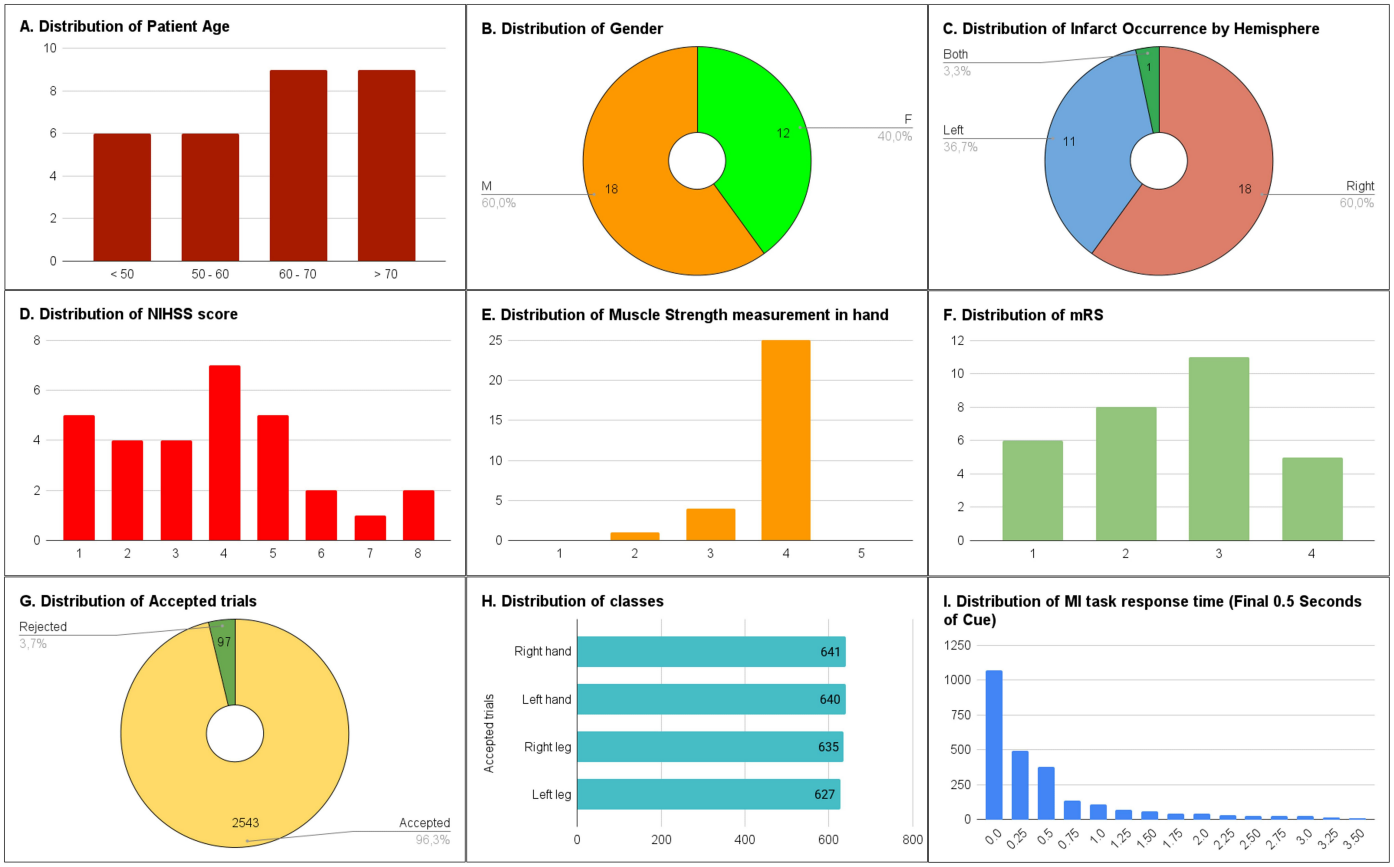
Metrics describing the UET175 dataset. **(A)** histogram showing distribution of patient age; **(B)** pie chart showing percentage of each genders; **(C)** pie chart showing distribution of infarct occurrence by hemisphere; **(D)** histogram showing distribution of NIHSS score; **(E)** histogram showing distribution of muscle strength measured in hand; **(F)** showing distribution of mRS score; **(G)** pie chart showing percentage of accepted trials after preprocesing; **(H)** bar chart showing classes distribution in the dataset; **(I)** histogram showing distribution of patient's response time measured from final 0.5 s of cue.

The completed dataset includes 220 sessions from 30 subjects. Each of these sessions contains at least one recording run as describe in the **Data Directory Structure**. Metrics describing the dataset are summarized in Figure 2. The stroke patients were between 43 to 78 years of age, with gender distribution of 40% female and 60% male (average 63.85, SD 12.85; Figures 2A, B). An initial analysis of the physician reports reveals a wide range of medications and medical conditions. All patients experienced an infarct, with the majority (62.1%) affecting the right hemisphere. However, only one patient exhibited an infarct that extended across both hemispheres (Figure 2C). The most common listed medical condition is damage to the corona radiata after stroke (26.7% of the medical reports included the text string “corona radiata”). Beside that, two of the most common underlying diseases among 30 patients are hypertension (80%) and diabetes mellitus type 2 (33.3%).

All patients had NIHSS scores ranging from 1 to 8, with the highest count of seven patients at a score of 4 (Figure 2D). Figure 2E reveals a strong skew toward a score of 4, suggesting that the majority of patients exhibit relatively high muscle strength in the Oxford scale, with very few having lower scores. However, despite the high Oxford score, the mRS scale indicates that most patients need assistance with daily tasks, with 11 patients at a moderate disability level and 5 at a moderately severe disability level (Figure 2F).

The average number of sessions per patient was 1.43, although as many as three sessions were recorded for a single patient over a 3-day period. Out of 220 sessions and 2,640 trials, only 97 trials (3.7%; Figure 2G) were rejected due to noisy signals and the lack of a significant ERD. This suggests that, despite using a commercial-grade EEG device, recording in a medical environment significantly enhances the quality of the recorded EEG signals. The accepted trials show similar distributions across all four classes (average 635.75 trials per class, SD 6.39; Figure 2H). Details analysis show that majority of the patient's responses to a MI task occurred within the last 0.5 s of cue, with a sharp decline thereafter (Figure 2I).

The UET175 dataset has been released and is freely available online at *https://github.com/nmk-k66-uet/UET175.git*. The uncompressed dataset comprise 434 MB, with a median Raw signal filesize of ~2 MB.

## Discussion

The UET175 dataset is novel because it addresses a significant gap in existing research: there has not been a dedicated database that focuses on EEG signals related to MI tasks in Vietnamese stroke patients and the use of more affordable, commercial-grade EEG devices. This lack data has hindered efforts to research and develop effective BCI systems tailored for this population. UET175 not only offers a valuable resource of EEG data, but also creates new avenues for interdisciplinary research. This can improve our understanding of the brain's mechanisms in MI-related contexts among stroke patients, ultimately fostering advances in rehabilitation and medical technology.

The data acquisition process for the UET175 dataset adheres to stringent standards to uphold ethical principles and security protocols. Before data collection begins, all patients are thoroughly informed about the research objectives, the procedures involved, and their rights, and they sign an informed consent agreement. Data is collected in a secure environment and rigorously protected to prevent any breaches of personal information. Additionally, security measures such as encryption and restricted access are implemented to ensure that all sensitive data is safeguarded and used exclusively for legitimate research purposes. Thus, UET175 not only serves as a valuable data source but also demonstrates a commitment to protecting the rights and safety of participating patients.

The UET175 dataset has been meticulously pre-processed to support research in machine learning and deep learning. This preprocessing involves noise removal, data normalization and segmentation, all designed to enhance the quality and accuracy of the information. The processed data can then be utilized to develop BCI systems for medical applications, aiming to improve rehabilitation outcomes for patients.

## Data Availability

The datasets presented in this study can be found in online repositories. The names of the repository/repositories and accession number(s) can be found at: https://github.com/nmk-k66-uet/UET175.
